# Lithium Toxicity: A Case Report

**DOI:** 10.1192/bjo.2025.10721

**Published:** 2025-06-20

**Authors:** Iqra Ain Ali, Imtiaz Ahmed Dogar

**Affiliations:** Faisalabad Medical University, Faisalabad, Pakistan

## Abstract

**Aims:** A 46-year-old female with a 30-year history of bipolar disorder presented with muscle stiffness, slurred speech, and altered sensorium, following fever, vomiting, and diarrhoea. She had been on lithium (400 mg daily) without regular monitoring. Examination showed confusion, hyperreflexia, tachycardia, and dehydration. Laboratory results revealed elevated serum lithium (3.4 mEq/L), renal dysfunction, hypernatremia, and echogenic kidneys. The diagnosis of lithium toxicity with acute kidney injury and dehydration-induced impaired excretion was confirmed. After discontinuing lithium, she underwent haemodialysis, and her condition improved. She developed lithium-induced diabetes insipidus, and long-term monitoring is required.

**Methods:** Case report.

**Results:** This case highlights the complexities of chronic lithium toxicity, presenting with neurological, systemic, and renal symptoms. Lithium accumulation exceeds renal clearance, particularly in the presence of factors like dehydration and acute kidney injury (AKI), leading to elevated serum lithium levels (3.4 mEq/L). The patient, with a history of bipolar disorder and long-term lithium use, developed classic neurological signs, including altered sensorium, tremors, hyperreflexia, and hypertonia, along with systemic manifestations such as anaemia, elevated AST, and abdominal symptoms. Lithium-induced nephrogenic diabetes insipidus (NDI) was confirmed, with persistent hypernatremia and polyuria despite normalized lithium levels.

Management included immediate discontinuation of lithium, hydration with intravenous Ringer’s lactate, and two sessions of haemodialysis, which effectively reduced lithium levels. Empirical ceftriaxone addressed a suspected infection, and quetiapine was initiated for mood stabilization. Long-term monitoring, including regular serum lithium and renal function checks, is crucial for patients on chronic lithium therapy, especially those with risk factors.

The case emphasizes the need for therapeutic drug monitoring, patient education on hydration, and early toxicity recognition. Despite clinical improvement, the patient’s prognosis remains guarded due to chronic damage from prolonged lithium exposure, including persistent NDI and hypernatremia.

**Conclusion:** Chronic lithium toxicity remains a preventable yet potentially life-threatening condition. Early recognition, regular monitoring, and timely intervention are paramount in mitigating its systemic, neurological, and renal effects. This case serves as a reminder of the importance of patient education, family involvement, and coordinated care to improve outcomes in bipolar affective disorder patients on lithium therapy.

